# Occupational radiation exposure from handheld dental x‐ray devices: A quantitative dosimetric study

**DOI:** 10.1002/acm2.70375

**Published:** 2025-11-18

**Authors:** Rawezh Ismael Abubakr, Shereen Ismail Hajee

**Affiliations:** ^1^ Medical Physics Unit Department of Pharmacology Medical Physics and Clinical Biochemistry College of Medicine Hawler Medical University Erbil Kurdistan Region Iraq; ^2^ Department of Dental Health Hawler Specialized Center for Oral and Dental Health Erbil Kurdistan Region Iraq

**Keywords:** dosimetry, handheld dental x‐ray, occupational safety, personal dose equivalent [Hp(d)], radiation exposure

## Abstract

**Background:**

Occupational radiation exposure from handheld dental radiographic devices may pose potential health risks. This study aims to quantitatively evaluate the occupational radiation dose from handheld dental radiographic devices, UAX‐01 (Device A), HyperLight‐G (Device B), and EzRay Air P (Device C) to the operator's critical anatomical regions.

**Methods:**

An experimental dosimetric study was conducted using purposive sampling of three devices. Radiation was measured using calibrated TLD‐100 dosimeters placed on a mannequin (simulating an operator) and a head and neck phantom used only to simulate patient scatter. Dosimeters were placed at the orbital surface, neck (thyroid level), chest (thoracic level), index fingers, and pelvic surface (gonadal level) to measure personal dose equivalent [Hp(0.07), Hp(10)] with and without shielding. Additionally, in vivo clinical dose evaluation for device A over 2 months with dosimeters placed at the chest and finger level for dentist and assistant in Erbil, Kurdistan region of Iraq. Personal dose equivalent was analyzed using SPSS, with statistical significance set at *p* ≤ 0.05.

**Results:**

The results showed significant differences in unshielded personal dose equivalent across devices and anatomical regions (*p* ≤ 0.05). UAX‐01 had the highest mean exposure at most sites: right orbital surface (46.90 µSv), neck (thyroid level) (39.54 µSv), chest (thoracic level) (40.66 µSv), right hand (65.21 µSv), left hand (31.85 µSv), and pelvic surface (gonadal level) (58.65 µSv). The left orbit received the highest dose from HyperLight‐G (26.68 µSv). Protective shielding significantly reduced radiation exposure in nearly all regions and devices (*p* < 0.001), with reductions ranging from 32.25% to 66.90%. An exception was the chest (thoracic level) with HyperLight‐G, where the reduction was not significant (*p* = 0.184). Additionally, in vivo monitoring of device A (UAX‐01) over 2 months revealed a cumulative personal dose equivalent Hp(0.07) =  16.76 mSv to the finger (sensor‐holding hand) and Hp(10) = 1.98 mSv to the chest of the dentist, indicating substantial localized exposure during routine clinical use.

**Conclusion:**

The study revealed that handheld dental x‐ray devices—particularly the UAX‐01—can expose operators to radiation levels that may exceed ICRP and NCRP occupational dose limits for the orbital surface, hands, and whole body when used without consistent protection. To mitigate these risks, health care providers should implement shielding, proper positioning, and sensor holders, while policymakers must enforce ALARA (as low as reasonably achievable)‐based regulations and routine occupational dose monitoring.

## 1 INTRODUCTION

Ionizing radiation is widely used in dentistry for diagnosis, prognosis, and therapeutic purposes. Dental radiographs are essential for detecting caries, identifying orofacial pathology, and evaluating dental development. While conventional dental radiographic systems are fixed and wall‐mounted, handheld alternatives have gained popularity over the past two decades.^1^, ^2^ The first handheld radiographic devices were developed for military use in the early 1990s and have since found diverse applications in dentistry, including mobile clinics, care for patients with reduced mobility, nursing homes, autopsies, morgues, and remote underserved regions. Unlike conventional fixed units, handheld devices are operated without consistent access to structural shielding, increasing the potential for occupational exposure. Despite their utility, the close proximity of the operator raises significant concerns regarding occupational radiation safety.^3^ Regulatory and safety authorities universally emphasize the ALARA principle, which mandates minimizing radiation exposure through effective time management, maintaining distance, and providing appropriate shielding.^3^, ^4^, ^5^

In 2015, the European Academy of Dento‐Maxillofacial Radiology issued guidelines for the use of handheld devices during treatments under general anesthesia, specifically for patients who require special care, in prisons, and in areas with limited access to dental facilities.^6^ Most handheld devices operate at a fixed 60 kV and 2.3 mA, featuring a focal spot of 0.4 mm and a source‐to‐skin distance of 20 cm.^7^ However, some units are inexpensive, lack essential safety mechanisms, and may not comply with U.S. Food and Drug Administration standards.^8^ Recent updates from the ICRP and NCRP highlight growing concern over increased use of handheld x‐ray devices in clinical settings and recommend stricter adherence to shielding protocols to remain within occupational dose limits.

Unlike fixed radiographic systems that operate within a controlled area, handheld dental x‐ray devices (HHDXDs) are used in proximity to the operator, exposing them to scatter and leakage radiation. Despite integrated shielding, this proximity increases the risk of occupational radiation exposure.^9^ To minimize exposure to the primary x‐ray beam, staff and the public are advised to maintain a minimum distance of 2 m from the radiation source. Effective radioprotection measures can significantly reduce the operator's effective dose. Optimal protection from secondary radiation is achieved when HHDXDs are set up on a stand and operated from a shielded area or a safe distance, similar to conventional radiographic systems.^10^, ^11^, ^12^ Long‐term radiation exposure can lead to deterministic effects, such as radiation‐induced cataracts, non‐malignant skin damage, fertility issues, and an increased risk of stochastic effects, including carcinogenesis and hereditary effects. The demand for handheld radiographic devices increased during the COVID‐19 pandemic, as they enabled rapid, point‐of‐care imaging while limiting equipment transfer and patient movement—particularly in high‐risk or infection‐controlled environments. This trend further underscores the importance of evaluating operator safety when using portable units.

Furthermore, growing concerns have been raised about radiation safety, particularly with the use of newly developed radiographic units. The International Commission on Radiological Protection (ICRP) recommends an annual occupational effective dose limit of 20 mSv, averaged over 5 years, while the National Council on Radiation Protection and Measurements (NCRP) recommends an annual effective dose limit of 50 mSv.^13^, ^14^ These limits aim to prevent deterministic outcomes and reduce the probability of stochastic effects, such as cancer.^2^ However, there is a lack of regional data evaluating operator radiation doses during routine dental radiographic procedures, particularly in Erbil, Kurdistan region, Iraq. Therefore, the present study aimed to conduct a quantitative dosimetric assessment of occupational radiation exposure to critical anatomical regions among operators using handheld x‐ray devices. In addition, the study sought to raise awareness among dental professionals in Erbil about radiation safety practices in line with the ALARA principle.

## 2 MATERIALS AND METHODS

### 2.1 Study design and setting

A controlled laboratory‐based experimental design was employed for this at the Iraqi Atomic Energy Commission, Radiological and Nuclear Applications and Laboratories Directorate in Baghdad, Iraq, from October 2024 to April 2025, and in vivo clinical measures were conducted over two months in Erbil, Kurdistan region of Iraq, from February 2025 to March 2025. The study's objective was to quantitatively assess and compare occupational radiation exposure associated with the clinical use of handheld dental radiographic units. Focusing on scatter radiation personal dose equivalent to the selected anatomical organs and tissues of operators with and without protection shielding.

### 2.2 Sampling technique

A purposive sampling technique using three HHDXDs commonly used in clinical practice across Erbil, Kurdistan region. This approach ensured the inclusion of units that reflect real‐world operator exposure conditions.

### 2.3 Handheld dental radiographic devices

**FIGURE 1:**
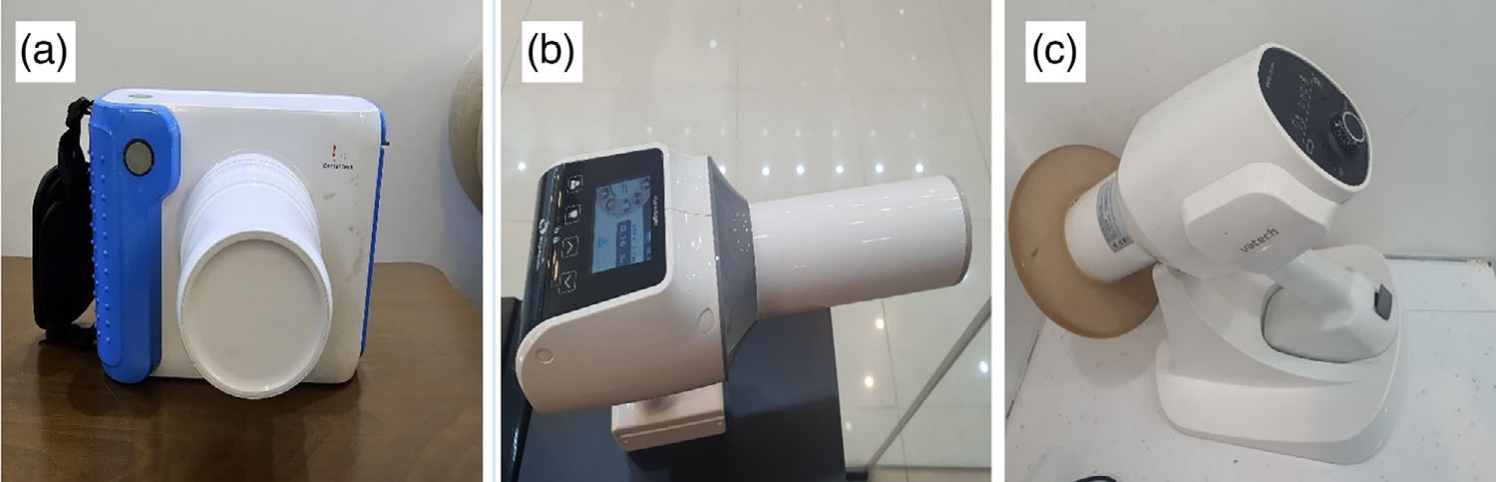
Handheld dental x‐ray devices.

Three commercially available HHDXDs were evaluated in this study. These devices were evaluated based on their operational parameters and ergonomic characteristics, and were used to irradiate thermoluminescent dosimeters (TLDs) strategically placed to simulate operator exposure. Technical specifications are detailed below and illustrated in Figure 1:

1. Device A–UAX‐01 (Foshan UAdenta Medical Equipment Co., Ltd, Foshan, Guangdong, China) operates at 60 kVp, 2 mA with 1 mm aluminum filtration, and a 0.4 mm focal spot size. The device weighs 2.2 kg, has a camera‐style design that requires two‐handed operation, and allows exposure time adjustment from 0.01 to 2 s via digital timer.
2. Device B–HyperLight‐G (Changzhou Sifary Medical Technology Co., Ltd., Changzhou, Jiangsu, China), operated at 65 kVp, 2 mA, included ≥ 1.8 mm aluminum filtration, and 0.4 mm focal spot size. Weighing 1.8 kg, it features a gun‐style ergonomic design for one‐handed use and supports exposure time settings between 0.02 and 2 s.
3. Device C–EzRay Air P (Vatech Co., Ltd., Hwaseong‐si, Gyeonggi‐do, South Korea), operates at 65 kVp, 2.5 mA, with a minimum of 1.5 mm aluminum filtration, and 0.4 mm focal spot. This device weighs 1.8 kg, features internal and external backscatter shielding, and supports one‐handed operation with an adjustable exposure time range from 0.05 to 1 s. Figure 1.

### 2.4 Phantom simulation

**FIGURE 2:**
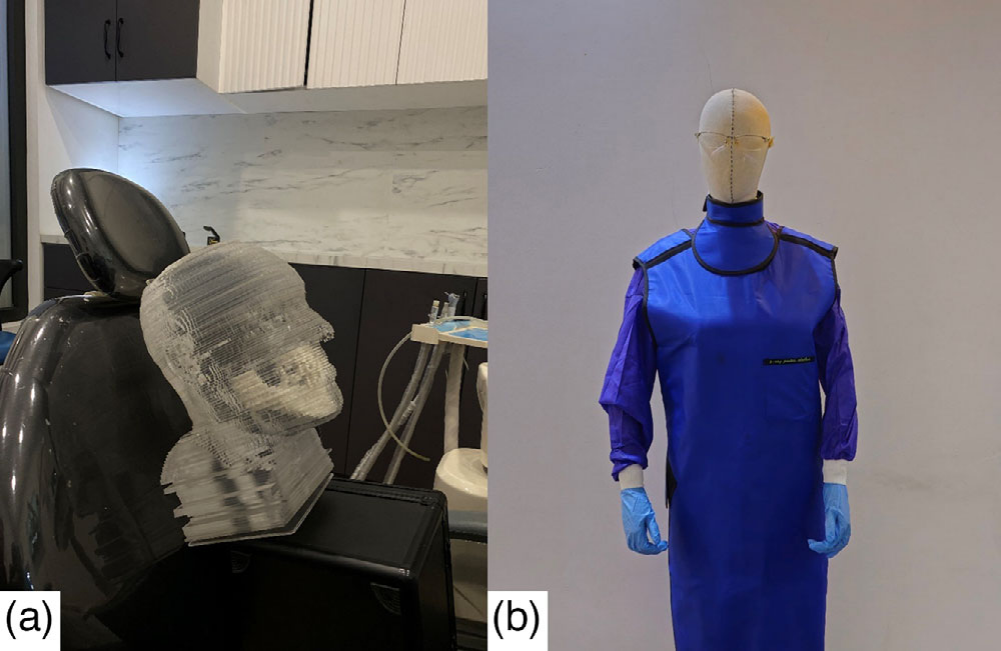
Experimental setup for dosimetric assessment. (a) Patient‐specific head and neck phantom constructed from urethane‐based resin and calcium carbonate, simulating bone and soft tissue properties for scatter radiation analysis. In addition, (b) a life‐sized operator mannequin positioned for exposure measurement, wearing full protective gear including lead apron, thyroid collar, leaded glasses, and lead gloves.

A patient‐specific head and neck dosimetry phantom was employed to simulate realistic scattering conditions during operator radiation exposure assessment. The phantom was constructed from a urethane‐based resin to mimic soft tissue properties, with calcium carbonate (CaCo_3_) added to simulate bone attenuation. Comprising 116 axial sections of 2.5 mm thickness, its anatomical structures were defined based on a standard human atlas to reflect radiological characteristics^15^, as shown in Figure 2.

### 2.5 Thermoluminescence dosimeter placement

TLD‐100 TLDs (Li:Mg,Ti) encapsulated in 7 mm/cm^2^ disposable vinyl pouches (Harshaw, Thermo Fisher Scientific Inc., USA) were used for dosimetric measurement. These dosimeters are highly sensitive to low‐energy photons and are commonly used for x‐ray dosimetry.^16^ TLDs were affixed to a life‐sized mannequin (165 cm tall) positioned to simulate a dental operator. The mannequin was placed 30 cm behind and slightly lateral to the head‐and‐neck phantom, oriented in a standard frontal clinical stance, positioned slightly behind and lateral to the phantom to replicate the operator's typical location during handheld dental radiography, with its face directed toward the phantom. Dosimeters were placed at five anatomical locations: right and left orbit (orbital surface), Neck (thyroid level), mid‐sternum chest (thoracic level), index fingers, and pelvic surface (gonadal level). Based on tissue depth and anatomical site, two types of dosimeters were used: TLD‐100 card dosimeters to measure deep dose equivalent, Hp(10), and TLD‐100 chip dosimeters to measure shallow dose equivalent, Hp(0.07). In accordance with international standards (ISO 15382:2015; ICRU Report 95; ICRP Publications 103), operational dose quantities were assigned to each anatomical site as follows: chest/neck at the thyroid level, and pelvic‐surface measurements were reported as Hp(10) [personal dose equivalent at 10 mm; deep‐dose monitoring used as a conservative surrogate for whole‐body and deep‐tissue exposure, including Neck (thyroid level), chest (thoracic level), and pelvic surface (gonadal level)]. Finger/hand ring TLDs and orbital surface chips were reported as Hp(0.07) (personal dose equivalent at 0.07 mm; shallow/skin or extremity dose). Lens‐of‐eye monitoring [Hp(3)] was not performed. We report shallow personal dose equivalent at the orbital surface [Hp(0.07)]; these data do not quantify lens‐of‐eye dose.^13^, ^17^, ^18^, ^19^, ^20^

The estimated unshielded Hp(10) values, measured at chest level, were used to approximate potential whole‐body exposure in the absence of protective equipment. Although this does not represent true organ‐specific effective doses, such operational quantities are commonly used for conservative dose monitoring in occupational settings.

Each device was used to perform a full‐mouth intraoral radiographic series comprising 16 exposures (seven maxillary periapical, seven mandibular periapical, and two bitewings), and the x‐ray tube angulation was adjusted accordingly to simulate real clinical scenarios. The mannequin remained stationary throughout all exposures to maintain consistent operator geometry, while the handheld x‐ray device was repositioned for each projection. Exposures were initiated remotely using a custom‐built 2‐m wooden extension arm.

### 2.6 Radiographic imaging protocols and TLD processing

**FIGURE 3:**
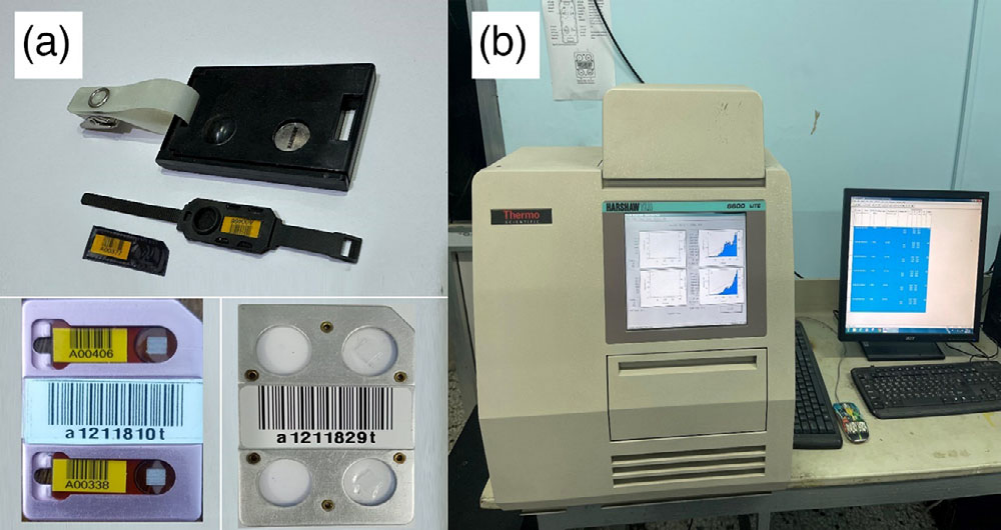
Dosimetry tools and processing equipment. (a) Thermoluminescent dosimeters (TLDs) and ring dosimeters used for measuring occupational radiation exposure, including chest‐level TLD badges and finger‐mounted EXT‐RAD rings. In addition, (b) Harshaw TLD Model 6600 automated reader system (Thermo Fisher Scientific) was used for reading and analyzing TLDs post‐exposure.

Each HHDXD was used to perform a standardized full‐mouth intraoral radiographic series comprising 16 exposures: two bitewing radiographs (right, left), seven maxillary periapical radiographs, and seven mandibular periapical radiographs.^21^ A digital intraoral sensor based on complementary metal oxide semiconductor (CMOS) technology was used in the setup. Device settings were UAX‐01 device (60 kVp, 2 mA,0.35 s), HyperLight‐G (65 kVp, 2 mA, 0.16 s), and EzRay Air P (65 kVp, 2.5 mA, 0.14 s). These settings are based on typical clinical use and manufacturer settings. Following exposure, TLDs were processed using an automated TLD reader (Harshaw Model 6600, Thermo Fisher Scientific) as illustrated in Figure 3. TLDs were calibrated using a radioactive source, Strontium‐90 (Sr‐90), which is built into the reader device at the Iraqi Atomic Energy Commission, ensuring traceability to national calibration standards. Quality control procedures included routine batch calibration checks and the use of control dosimeters to correct for environmental background. Reproducibility of TLD measurements was evaluated using three times exposures, with a coefficient of variation (CV) below 7%.

### 2.7 Data management and statistical analysis

Data were analyzed using the Statistical Package for Social Sciences (SPSS), version 26 (IBM Corp., Armonk, New York, USA). Normality of the data was assessed using the Shapiro–Wilk test, which indicated that the variables were normally distributed. Therefore, we adhered to parametric statistical tests for subsequent analyses. An unpaired *t*‐test was used for comparisons between two independent groups, while a one‐way analysis of variance (ANOVA) was applied to compare the means of the three devices, followed by least significant difference (LSD) post hoc testing to examine pairwise group differences. Pairwise post hoc comparisons were conducted using the LSD test, given the limited number of groups (three devices) and the predefined pairwise comparisons. Planned pairwise contrasts among the three devices (A vs. B, A vs. C, B vs. C) yielded 21 pre‐specified comparisons across seven anatomical regions. A *p* value of ≤ 0.05 was considered statistically significant.

### 2.8 Ethical approval and informed consent

This study adhered to the guidelines of the Institutional Research Ethics Board and the Declaration of Helsinki. Ethical approval was obtained from the Research Ethics Committee of the College of Medicine, Hawler Medical University, Kurdistan Region of Iraq (Approval code: meeting code: 1, paper code: 6, dated September 22, 2024). Oral informed consent was obtained from participants prior to in vivo radiation monitoring. All personal identifiers were removed, and the data were anonymized prior to analysis.

## 3 RESULTS

This study evaluated the unshielded scatter radiation personal dose equivalent to dental operators across key anatomical regions. Background radiation was measured using TLDs, not included in mannequin measurements, and it was 78.88 and 143 µSv. Radiation dose measurement was conducted at specific anatomical regions of the operator: right orbit, left orbit, neck (thyroid level), chest (thoracic level), right hand (index finger), left hand (index finger), and pelvic surface (gonadal level). All exposures were conducted under unshielded conditions, repeated three times for each anatomical region and device, and the averaged values are presented in Table 1.

**TABLE 1:** Mean unshielded personal dose equivalent (µSv) by anatomical region and device.

Anatomical regions	Device A(UAX‐01)^a^	Device B (HyperLight‐G)^a^	Device C (EzRay Air P)^a^	*p* ^*^
Right orbit	46.90 ± 14.32	31.34 ± 8.14	16.63 ± 3.26	< 0.001
Left orbit	22.62 ± 4.53	26.68 ± 7.94	19.43 ± 3.17	0.003
Neck (thyroid level)	39.54 ± 10.44	20.47 ± 7.55	29.76 ± 6.78	< 0.001
Chest (thoracic level)	40.66 ± 13.36	28.43 ± 14.21	27.86 ± 4.61	0.004
Right hand	65.21 ± 19.71	61.34 ± 11.74	42.45 ± 9.76	< 0.001
Left hand	31.85 ± 7.8	30.8 ± 8.24	14.89 ± 2.99	< 0.001
Pelvic (gonadal level)	58.65 ± 16.82	49.05 ± 12.16	35.82 ± 13.28	< 0.001

Chest, neck, and pelvic‐surface reported as Hp(10); hands and orbit reported as Hp(0.07).

^a^ Values are mean ± SD.

* Calculated by one‐way ANOVA.

There were statistically significant differences in unshielded mean personal dose equivalent (µSv) across all anatomical regions when using the three HHDXDs (*p* < 0.05). Device A (UAX‐01) produced the highest radiation exposure in most regions, including the right orbit (46.90  ±  14.32 µSv, *p* < 0.001), neck (thyroid level) (39.54  ±  10.44 µSv, *p* < 0.001), chest (thoracic level) (40.66  ±  13.36 µSv, *p* = 0.004), right hand (65.21  ±  19.71 µSv, *p* < 0.001), and pelvic surface (gonadal level) (58.65  ±  16.82 µSv, *p* < 0.001). The left orbit showed a significant difference as well (*p* = 0.003), with Device B having the highest dose (26.68  ±  7.94 µSv). The left hand also showed significant variation among devices (*p* < 0.001), with Device C recording the lowest dose (14.89  ±  2.99 µSv). Overall, Device C (EzRay Air P) consistently demonstrated the lowest exposure across most anatomical regions, while Device B (HyperLight‐G) showed intermediate values (Table 1).

The results showed that pairwise comparisons of mean unshielded personal dose equivalent revealed significant differences between devices for most anatomical regions. Device A showed significantly higher doses than both Device B and Device C in the right orbit, neck (thyroid level), and chest (thoracic level) regions (*p* < 0.01). For the left orbit, Device B produced higher exposure than Device C (*p* = 0.001), while the difference between Device A and B was marginally significant (*p* = 0.046), and A versus C was not statistically significant (*p* = 0.112). No significant difference was found between Device A and B for the right (*p* = 0.450) and left hand (*p* = 0.663), although Device C showed significantly lower doses in both hands (*p* < 0.001). For the pelvic surface (gonadal level), Device A versus C (*p* < 0.001) and Device B versus C (*p* = 0.012) were significant, while A versus B was not (*p* = 0.063) (Table 2).

**TABLE 2:** Pairwise comparisons of mean unshielded personal dose equivalent by anatomical region.

Anatomical regions	Device A vs. B^a^	Device A vs. C^a^	Device B vs. C^a^
Right orbit	< 0.001	< 0.001	< 0.001
Left orbit	0.046	0.112	0.001
Neck (thyroid level)	< 0.001	0.002	0.003
Chest (thoracic level)	0.005	0.003	0.890
Right hand	0.450	< 0.001	0.001
Left hand	0.663	< 0.001	< 0.001
Pelvic (gonadal level)	0.063	< 0.001	0.012

^a^ One‐way ANOVA with LSD post hoc (planned pairwise contrasts among devices). Reported values pertain to Hp(10) for chest/neck/pelvic‐surface and Hp(0.07) for hands/orbital surface.

Subsequently, the effect of protective shielding on radiation exposure was evaluated. The mean personal dose equivalent measured using standard protective measures, including lead apron, thyroid collar, leaded glasses, and lead gloves, was associated with a statistically significant reduction in radiation exposure across nearly all anatomical regions (*p* ˂ 0.05), as determined by an unpaired *t*‐test. The only exception was the chest (thoracic level) region in HyperLight‐G (*p* = 0.184), as illustrated in Table 3, along with corresponding percentage reductions.

**TABLE 3:** Mean personal dose equivalent (µSv) under unshielded/shielded conditions.

Anatomical regions	UAX‐01	*p* ^*^	HyperLight‐G	*p* ^*^	EzRay Air P	*p* ^*^
Right orbit	40.69/13.47	<0.001	31.34/12.43	<0.001	16.63/8.57	<0.001
Left orbit	22.62/7.9	<0.001	26.68/12.33	0.001	19.43/10.36	<0.001
Neck (thyroid level)	39.54/14.48	<0.001	20.47/12.61	0.039	29.76/19.53	<0.001
Chest (thoracic level)	40.66/19.95	0.003	28.43/19.26	0.184	27.86/16.47	<0.001
Right hand	65.21/39.56	<0.001	61.34/32.85	<0.001	42.45/20.42	<0.001
Left hand	31.85/21.19	0.009	30.80/ 14.68	0.001	14.89/9.31	0.001
Pelvic (gonadal level)	58.65/35.10	<0.001	49.05/ 29.73	0.003	35.82/21.38	0.030

Personal dose equivalent reported as Hp(10) (chest/neck/pelvic‐surface) or Hp(0.07) (hands/orbital surface).

* Unpaired *t*‐tests compare unshielded vs. shielded within device/region.

**FIGURE 4:**
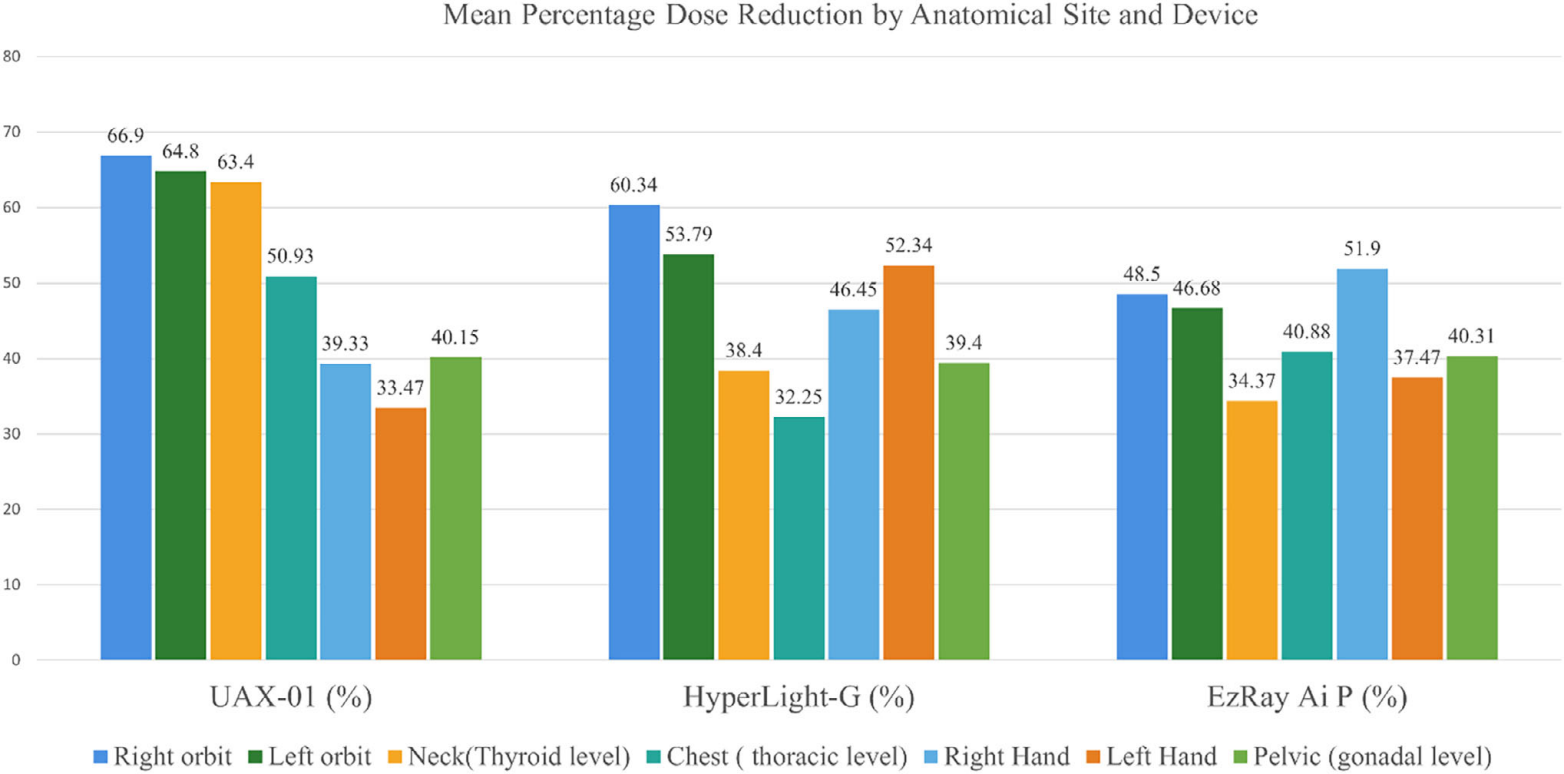
Mean dose reduction unshielded/shielded across key operators' anatomical regions and devices in µSv.

As shown in Table 3 and Figure 4, the application of personal protective equipment (PPE) significantly reduced personal dose equivalent across all anatomical region, the most pronounced reduction were observed in the orbital surface and neck (thyroid level), particularly with device A, which obtained reduction exceeding 63% statistically significant (*p* ˂ 0.001), moderate reduction were seen in chest (thoracic level), and pelvic surface (gonadal level) across three devices with *p* values ranging from 0.003 to 0.001, underscoring protective value of lead apron. while the hand represented variable reduction, with device C showing the substantial attenuation in the right hand (51.90%, *p* ˂ 0.001) and device B in the left hand (52.34%, *p* = 0.001), this variation may reflect ergonomic differences in device design, operator positioning, or shielding consistency.

### 3.1 Estimated thoracic Hp(10) as a surrogate for whole‐body exposure and comparison with occupational limits (unshielded condition)

Thoracic Hp(10) was measured at the chest (personal dose equivalent at 10 mm) and is used here as a conservative indicator of whole‐body exposure under unshielded conditions, consistent with international monitoring practice. Hp(10) is an operational monitoring quantity rather than an organ dose or ICRP‐defined effective dose; results are interpreted accordingly. To evaluate cumulative occupational radiation exposure, we report the annualized thoracic Hp(10) measured at the chest (personal dose equivalent at 10 mm). Under unshielded conditions, thoracic Hp(10) is used as a conservative indicator of whole‐body exposure. Measurements at the neck (thyroid level) and pelvic surface are likewise reported as Hp(10). The estimation assumed a clinical workload of 100 exposures per week, over 46 working weeks per year, resulting in a total of 4600 exposures annually.^22^

Consistent with ICRP/ICRU guidance, we did not derive ICRP‐defined effective dose (E) from single‐site TLD measurements or apply tissue‐weighting factors to Hp(10). Organ‐specific doses and *E* would require suitable conversion methodology (e.g., coefficients from ICRP Publication 116) or dedicated phantom/Monte Carlo simulations. As surface measurements depend on irradiation geometry, operator positioning, and the scattered field, Hp(10) can overestimate or underestimate organ doses; therefore, these values should be interpreted strictly as operational surrogates.

The annualized Hp(10) values at the neck (thyroid level), chest (thoracic level reported as a surrogate for lung exposure), and pelvic surface (reported as a surrogate for gonadal exposure) are reported in Table 4 and compared with ICRP and NCRP occupational dose limits. For whole‐body exposure, the ICRP recommends an average of 20 mSv/year over 5 years (not exceeding 50 mSv in any single year), while NCRP 116 recommends a limit of 50 mSv/year.^14^, ^21^ NCRP 116 also specifies an equivalent dose limit of 500 mSv/year for the thyroid, which is provided here for context. The sum of Hp(10) values measured at the neck, chest, and pelvic surface suggests that cumulative annual exposures could exceed ICRP limits under unshielded conditions.

**TABLE 4:** Annual Hp(10) at neck (thyroid level), chest (thoracic level), and pelvic surface (gonadal level) under unshielded conditions.

Measurement site [Hp(10)]	Device	Hp(10) (mSv/year)	ICRP Limit (mSv/year)	NCRP (mSv/year)
Neck (thyroid level)	A	181.88	–	500
	B	94.16	–	500
	C	136.9	–	500
Chest (thoracic level)	A	187	20	50
	B	130.75	20	50
	C	128.17	20	50
Gonad (pelvic surface)	A	269.75	20	50
	B	225.63	20	50
	C	164.75	20	50

*Note*: Hp(10) values represent personal dose equivalent measured at the neck (thyroid level), chest (thoracic level, surrogate for lung exposure), and pelvic surface (gonadal level).

### 3.2 Estimated Hp(0.07) for eye (orbital surface), hands, and comparison with occupational limits (unshielded)

According to ICRP publication 118 (2012), the occupational equivalent limit dose to the eye's lens is 20 mSv per year, averaged over 5 years with no individual year exceeding 50 mSv. In ICRP 103, equivalent dose limits to the eye were recommended as 150 mSv/year and 500 mSv for the hand.^13^, ^23^

In this study, Hp (0.07) dosimeters were used to measure radiation exposure to the eyes and hands, representing shallow dose equivalent at 0.07 mm tissue depth. As summarized in Table 5, all three evaluated devices recorded annual eye doses (right and left) that exceeded the current occupational limit of 20 mSv/year set by ICRP 118. Notably, Device A's eye dose readings also surpassed the older threshold of 150 mSv/year specified in ICRP 103, indicating a significant risk of overexposure in the absence of protective measures. Although Hp(3) was not measured, orbital Hp(0.07) may over‐ or underestimate the lens dose; therefore, comparisons with lens‐dose limits (ICRP 118) are indicative only and require confirmation with dedicated Hp(3) dosimetry.

**TABLE 5:** Estimated annual personal dose equivalent Hp(0.07) for orbital and hands (Unshielded).

Device	Body site	Annual Hp(0.07) (mSv/year)	ICRP Limit 103 (mSv/year)	ICRP Limit 118(mSv/year)	NCRP 116(mSv/year)
Device A	Right orbit	215.74	150	20	150
	Left orbit	104.052			
Device A	Right hand	299.97	500	500	500
	Left hand	146.51			
Device B	Right orbit	144.16	150	20	150
	Left orbit	122.73			
Device B	Right hand	282.16	500	500	500
	Left hand	141.68			
Device C	Right orbit	76.50	150	20	150
	Left orbit	89.38			
Device C	Right hand	195.27	500	500	500
	Left hand	68.49			

Orbital Hp (0.07) was used as a surrogate; the lens limit applies to Hp(3)/lens equivalent dose (ICRP 118). Thus, comparisons with the lens limit are indicative and should be interpreted with caution pending dedicated Hp(3) monitoring. All values are annualized, assuming 100 exposures/week for 46 weeks (4600/year).

In contrast, the personal dose equivalent Hp(0.07) to the hand for all devices remained well below the ICRP‐recommended occupational limit of 500 mSv/year,^13^ as summarized in Table 5.

### 3.3 In vivo cumulative scattered radiation dose monitoring using thermoluminescent dosimeters (TLDs) and ring dosimeters

During routine clinical use of handheld dental radiographic units, occupational radiation exposure was monitored over a 2‐month clinical period using device A (UAX‐01). Dosimetry was performed using a Harshaw TLD‐100 card dosimeter (chest) and EXT‐RAD ring dosimeters (dominant hand), Figure 3. Two operators were evaluated: a dentist who manually held the sensor during exposure and a dental assistant who activated the device. Due to logistical limitations in acquiring additional dosimeters from the Iraqi Atomic Energy Commission, the Radiological and Nuclear Application and Laboratory Directorate was unable to conduct tests on other devices, and the results are summarized in Table 6.

**TABLE 6:** Cumulative occupational radiation dose over 2 months during clinical practice (mSv), device A.

Operator	Anatomical region and dosemeter type	Dose type	Background dose (µSv)	Net Dose (mSv)
Dentist	Chest (TLD)	Hp(10) deep dose	274.92	1.6887
Dentist	Chest (TLD)	Hp(0.07) shallow dose	247.65	1.98477
Dentist	Finger (ring dosimeter)	Shallow dose	290.83	16.76138
Assistant	Chest (TLD)	Hp(10) deep dose	274.92	1.36502
Assistant	Chest (TLD)	Hp(0.07) shallow dose	247.65	1.73369
Assistant	Finger (ring dosimeter)	Shallow dose	290.83	3.42781

UAX‐01 device dosimetry was performed during clinical routine over 2 months. Net dose obtained after correction for background radiation.

## 4 DISCUSSION

The study aimed to quantitatively assess occupational radiation exposure among dental professionals using handheld dental radiographic devices, through both phantom‐based dosimetry and in vivo clinical monitoring, and to compare these exposures against established international guidelines. The findings revealed significant variation in radiation dose across devices, anatomical regions, and shielding conditions. This variability highlights the need for model‐specified evaluation in clinical settings. Among the devices evaluated, the UAX‐01 consistently generated the highest unshielded personal dose equivalent, followed by HyperLight‐G and EzRay Air P, which yielded the lowest exposure due to their enhanced internal shielding. Although the LSD test was used to enhance sensitivity given the limited number of comparison groups, we acknowledge that LSD does not adjust for multiple comparisons, which may increase the risk of Type I errors. Therefore, borderline significant findings should be interpreted with caution.

When compared against international radiation protection guidelines, several estimated personal dose equivalents exceeded occupational dose limits. For example, the personal dose equivalent to the orbit Hp(0.07), from the UAX‐01 device, was 215.74 mSv/year, which exceeds both the ICRP 118 limit of 20 mSv/year (averaged over five years) and the older ICRP 103 threshold of 150 mSv/year. Among all anatomical regions, the thoracic Hp(10)—reported as a surrogate for lung exposure—showed the highest annualized value (187 mSv/year for Device A, as presented in Table 4), which substantially exceeds the ICRP occupational dose limit of 20 mSv/year (averaged over 5 years). Pelvic Hp(10) values were also elevated (269.75, 225.63, and 164.75 mSv/year for Devices A, B, and C, respectively), although these represent operational monitoring quantities rather than true organ doses. These represent operational monitoring quantities and not true effective doses. Although hand exposure levels remained below the 500 mSv/year limit defined for extremities, Hp(0.07) to the right hand reached 299.97 mSv/year, representing a substantial localized burden, particularly during sensor holding. These values were derived using the operational quantities Hp(10) and Hp(0.07), as recommended by ICRP Publication 103 and ISO 15382. This standardization ensures that reported doses accurately reflect regulatory frameworks and occupational dose limits for stochastic and deterministic effects.^18^, ^24^

While the extrapolation to annual dose was based on a standardized workload of 100 exposures per week over 46 weeks per year (4600 exposures annually), this estimate may not reflect the full variability of clinical practice. Differences in patient volume, workflow efficiency, staff rotation, and device usage patterns across dental settings can significantly influence real‐world operator exposure. Moreover, certain practices may involve higher exposure frequencies due to procedural demand or lack of auxiliary personnel, further elevating the occupational risk. Therefore, while the extrapolated doses provide a valuable benchmark, they should be interpreted with caution, and further longitudinal monitoring across multiple centers is recommended to capture the full spectrum of occupational exposure in routine practice.

The findings highlight the urgent need for improved shielding practice and ergonomic device improvement. The UAX‐01, with a total filtration of 1 mm Al, generated the highest scatter personal dose equivalent at nearly all anatomical landmarks. HyperLight‐G exhibited an intermediate exposure value, whereas EzRay Air P, featuring improved shielding, resulted in the lowest operator dose. These findings illustrate the importance of device design and preventive measures in mitigating occupational radiation exposure. In vivo monitoring using the UAX‐01 device confirmed substantial radiation exposure during routine clinical use. In that assessment, the dentist who directly handled the sensor received a cumulative shallow dose Hp(0.07) of 16.76 mSv to the finger and a chest dose Hp(10) of 1.98 mSv over 2 months of routine use. If such exposure continued throughout the year, it could exceed the NCRP and approach or surpass limits for extremities, underscoring the need for mandatory use of sensor holders and PPE.

This study is particularly significant for the Kurdistan Region of Iraq, where handheld devices are frequently used without adequate shielding. Despite their portability and utility in outreach settings, their operator safety has not been adequately assessed. By integrating phantom‐based and in vivo dosimetry, this research addresses a critical knowledge gap and provides region‐specific data to support safer clinical practices.

The result of the present study aligns with Geist et al.'s recommendation to restrict use due to increased exposure risks and manual operation constraints.^6^ Complementing this, Kim et al. quantitatively demonstrated that handheld units produce significantly greater levels of leakage and scatter radiation than fixed radiographic units.^25^

Our findings are consistent with those of Altindag et al., who reported high unshielded doses, including right‐hand dose was 119.4 µGy for Rextar X and 71.7 µGy for Diox 602, and 31.8 µGy to the eye and 30.5 µGy to the thyroid. Their study also demonstrated significant shielding‐related dose reductions ranging from 11.49% to 93.25%, depending on the device and region. Similarly, our study confirms that the scattered personal dose equivalent from portable dental x‐ray devices contributes significantly to operator dose, specifically when the operator is positioned close to the device without structural shielding.^21^ The elevated occupational doses recorded in our study, specifically to the dominant hand and orbital level, align with the findings of Makdissi et al., who reported that operator exposure varied significantly depending on handling technique and proximity to the radiation sources.^22^ Although many handheld models incorporate built‐in shielding, our findings confirm that such measures are insufficient without the addition of external protection. Accordingly, we recommend the routine use of personal dosimeters, sensor holders, and remote triggering when operating near patients. Devices with built‐in backscatter shielding as per ISO 15382 guidelines.^26^ Ivanović et al. also emphasized the importance of proper positioning and protective protocols, which align with the present study's outcomes.^7^ Our findings are supported by Otaka et al. (2024), who demonstrated that using a rectangular collimator and detector holder during handheld x‐ray exposure reduced operator dose by up to 87%. Their study emphasizes that simple, low‐cost accessories can significantly limit stray radiation, aligning with our recommendation for enhanced shielding strategies in clinical practice.^27^

Moreover, Rottke et al. (2018) demonstrated that HHDXDs can expose operators to measurable doses, but proper alignment of the beam, shielding use, and increasing distance significantly reduce this risk. Their findings support our results, emphasizing that without consistent protective measures, occupational exposure may exceed recommended limits even with modern handheld units.^28^

In this study, the highest unshielded personal dose equivalent to the dominant(right) hand was 65.21 µSv with the UAX‐01 device, and the lowest value of shielded condition of the EzRay Air P device was 20.42 µSv. This variation can be attributed to differences in internal shielding and ergonomic configurations, which influence the operator's hand proximity to the x‐ray beam and how the operator grips the device. UAX‐01 device, which features a camera‐like design, requires the operators to grip the unit from both sides near the housing, potentially increasing scatter exposure compared to designs that allow greater hand distance or incorporate integrated shielding around the grip. When protective shielding was applied, marked reductions in hand dose were observed across all devices. For the right hand, the dose reduction was 39.33% with the UAX‐01 device, 46.45% reduction with HyperLight‐G, and 51.90% reduction with EzRay Air P. These findings show the effectiveness of localized hand shielding in reducing operator dose.

HHDXDs are typically operated at arm's length and parallel to the floor, placing the operator close to the radiation source. Unlike fixed or mobile systems, which rely on distance and shielding, handheld use often bypasses established “controlled area” protocols. This deviation increases the risk of occupational radiation exposure for dental professionals.^29^

In contrast to deterministic effects such as cataracts and skin injury, which occur above specific dose thresholds, stochastic effects like carcinogenesis follow the linear no‐threshold (LNT) model, where minimal doses of ionizing radiation can carry long‐term biological risks, particularly when exposures are repeated or compounded with other occupational sources. The ICRP affirms that no radiation dose is entirely without risk.^13^ This study's adherence to dose reporting using operational quantities Hp(10) and Hp(0.07) enhances its compliance with current international standards and improves the interpretability of occupational risk. The clinical monitoring of a single device and the lack of standardized operator positioning limited this study, potentially affecting dose variability. Additionally, the lack of standardization in operator positioning during phantom‐based procedures may have introduced variances in dose measurements. Further studies should incorporate long‐term monitoring to enhance the assessment of ergonomic design and shielding efficacy. Additionally, the in vivo component of this study was limited by a small clinical sample size, involving only two clinical operators and one handheld device (UAX‐01), due to logistical restrictions in acquiring sufficient dosimeters and access permissions. As such, the generalizability of the in vivo findings may be constrained, and the results should be interpreted with caution. Larger‐scale clinical studies involving multiple devices, operators, and longer observation periods are recommended to validate and extend these findings. The estimated unshielded Hp(10) values, measured at chest level, were used to approximate potential whole‐body exposure in the absence of protective equipment. Although this does not represent true organ‐specific effective doses, such operational quantities are commonly used for conservative dose monitoring in occupational settings. All device configurations exceeded the ICRP/NCRP recommended limit of 20 mSv/year when no shielding was applied, suggesting a significant exposure risk under unprotected conditions. Moreover, the absence of Hp(3) dosimetry prevented direct measurement of the lens dose of the eye, which may lead to under‐ or over‐estimation when extrapolating from Hp(0.07) measurements. Measurement accuracy may also be influenced by TLD variability and positioning errors, despite the implementation of quality assurance protocols. Finally, while our exposure setup simulated realistic clinical conditions, it remains a simplified model that may not fully capture variations in daily clinical workflows, operator movement, or environmental factors such as room geometry and backscatter from surrounding surfaces.

## 5 CONCLUSION

This study confirms that HHDXDs pose significant occupational radiation risks to operators, particularly to the hands and orbital level. In high‐workload clinical settings lacking consistent shielding, operator doses may exceed recommended limits. To mitigate exposure, adherence to the ALARA (As Low As Reasonably Achievable) and ALADA (As Low As Diagnostically Acceptable) principles is essential. The use of protective accessories—such as shielding discs, extended cones, sensor holders and routine PPE—should be strongly encouraged. Further real‐world clinical investigations are needed to assess cumulative exposures over time and to strengthen radiation safety protocols.

## AUTHOR CONTRIBUTIONS

**Rawezh Ismael Abubakr**: Conceptualization; data curation; formal analysis; investigation; methodology; visualization; writing—original draft; writing—review & editing. **Shereen Ismail Hajee**: Supervision; writing, review & editing.

## CONFLICT OF INTEREST STATEMENT

The authors declare no conflicts of interest.

## Data Availability

The data supporting the findings of this study are available from the corresponding author upon reasonable request.
